# Notch‐1 regulates collective breast cancer cell migration by controlling intercellular junction and cytoskeletal organization

**DOI:** 10.1111/cpr.13754

**Published:** 2024-09-29

**Authors:** Yixi Zhang, Xiang Qin, Ronghua Guo, Xiyue Sun, Zihan Zhao, Hanyu Guo, Meng Wang, Shun Li, Tingting Li, Dong Lv, Yiyao Liu

**Affiliations:** ^1^ Department of Pharmacy, Personalized Drug Therapy Key Laboratory of Sichuan Province, Sichuan Provincial People's Hospital, and School of Life Science and Technology University of Electronic Science and Technology of China Chengdu China; ^2^ Department of Urology Deyang People's Hospital Deyang China; ^3^ TCM Regulating Metabolic Diseases Key Laboratory of Sichuan Province Hospital of Chengdu University of Traditional Chinese Medicine Chengdu China

## Abstract

Pathological observations show that cancer cells frequently invade the surrounding normal tissue in collective rather than individual cell migration. However, general principles governing collective cell migration remain to be discovered. Different from individual cell migration, we demonstrated that the Notch‐1‐activation reduced collective cells speed and distances. In particular, Notch‐1‐activation induced cellular cytoskeletal remodelling, strengthened the intercellular junctions and cell‐matrix adhesions. Mechanistically, Notch‐1 activation prevented the phosphorylation of GSK‐3β and the translocation of cytoplasmic free β‐catenin to the nucleus, which increased E‐cadherin expression and tight intercellular junctions. Moreover, Notch‐1 signalling also activated the RhoA/ROCK pathway, promoting reorganization of F‐actin and contractile forces produced by myosin. Further, Notch‐1 activation increased cell adhesion to the extracellular substrate, which inhibited collective cell migration. These findings highlight that cell adhesions and cell–cell junctions contribute to collective cell migration and provide new insights into mechanisms of the modulation of Notch‐1 signalling pathway on cancer cell malignancy.

## Graphical abstract

Notch‐1 activation (NICD) inhibits collective cell migration by promoting high intracellular force and strong cell–cell adhesion. Notch‐1 activation leads to GSK‐3β activity by inhibiting ILK phosphorylation, β‐ catenin is degraded and cannot enter the nucleus, resulting in higher expression of E‐cadherin and tighter intercellular connections. Additionally, Notch‐1 stimulates the Rho/ROCK pathway, which enhances adhesions between cells and the extracellular matrix and ultimately impedes collective cell migration.

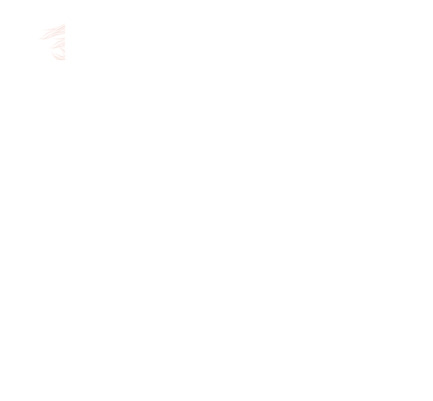

## 1 INTRODUCTION

In recent years, numerous studies have put forth the mechanisms underlying cancer metastasis, which enable tumour cells to migrate to distant sites through the reorganization and rebuilding of cytoskeleton.^1^ Notably, compared to individual cell migration, pathological tissue sections of cancer patients have demonstrated that certain types of tumour cells tend to converge and migrate collectively, a phenomenon known as collective cell migration.^2^ This process, which serves as the primary mode of cellular motility, plays a key role in several biological processes in the body, including angiogenesis, convergent extension and branching morphogenesis.^3^, ^4^, ^5^, ^6^ In collective cell migration, leader cells located at the forefront of cellular clusters extend lamellipodia, migrate to the free edge and subsequently pull along follower cells.^7^ These forces also trigger the reorganization of the actomyosin cytoskeleton, coordinating the polarity and velocity of migration.^8^, ^9^ Notably, mounting evidence supports the notion that carcinoma invasiveness and metastasis are closely linked to collective cell migration.^10^, ^11^ Therefore, a better understanding of the cellular signalling pathways regulating collective cell migration may help identify new therapeutic targets for invasive and late‐stage disease patients.

The interconnection between Notch signalling and collective cellular migration in neoplastic cells is a subject of immense interest and significance. The ubiquitous Notch signalling pathway in mammals regulates numerous biological processes, including cancer malignancy.^12^ It is triggered by the interaction of Notch ligands with adjacent Notch receptors expressed in neighbouring cells.^13^ Following binding, the Notch receptor undergoes sequential cleavage by the metalloprotease (ADAM) family metalloprotease and the γ‐secretase complex, resulting in the release of the Notch‐1 intracellular domain (NICD).^14^ The NICD relocates to the nucleus where it forms a transcriptional regulatory complex with other coactivators to govern downstream gene expression and modulate various cellular processes, including differentiation, proliferation and apoptosis. Recent research has suggested that after Notch‐1 activation, cells produce epithelial‐mesenchymal transition (EMT), which leads to the initiation of squamous cell carcinoma, which indicates that Notch‐1 signal can regulate the changes of cell morphology and movement.^15^, ^16^ Further studies have also shown that Dll4 signalling through both Notch‐1 and cellular stress dynamically regulates the leader cell identity in a migrating epithelium.^17^ Notch‐1 signalling was also found to regulate cell–cell junctions,^18^ which indicates the essential role of Notch‐1 in collective cancer cell migration.

Recent studies have utilized in vitro culture models to investigate the mechanisms of collective cell migration.^19^ However, our understanding of this process remains incomplete due to its inherent complexity, involving coordination between cells within biological tissues and the basement membrane involves mechanical and biochemical interactions,^5^ which may differ from individual cells.^20^, ^21^ Therefore, it is crucial to comprehend the reasons for collective cell movement instead of single cell migration and how individual cell movements are regulated to facilitate coordinated migration. Adhesion molecules, such as E‐cadherin, are continuously expressed to maintain cell–cell junctions and ensure orderly migration.^22^, ^23^, ^24^ Collective cells interconnect through junctional complexes, which consist of tight junctions (TJ), adherens junction (AJ, formerly zonula adherens) and a desmosome located at the apical‐most end of cell–cell contacts.^23^, ^25^, ^26^ E‐cadherin serves as a primary adhesion receptor at the AJ of epithelial cells.^26^ It binds to β‐catenin or plakoglobin, which then associates with αE‐catenin to form the cadherin‐catenin complex.^27^ αE‐catenin interacts with F‐actin in two ways: directly and indirectly through binding with vinculin. β‐catenin is a multifunctional protein that regulates cell adhesion and nuclear transcription.^28^ β‐catenin located on the cell membrane binds to E‐cadherin at the adhesion junction, which constitutes an indispensable component of intercellular adhesion and stability. β‐catenin present in the cytoplasm is tightly regulated by protein complexes consisting of axin, glycogen synthase kinase 3b (GSK‐3β) and adenomatous polyposis coli (APC). Consequently, the accumulated cytoplasmic β‐catenin translocates to the nucleus, thereby initiating its oncogenic functionalities. Several transcriptional repressors of E‐cadherin, including snail and slug, have recently been identified. Free β‐catenin may potentially partake in the stabilization of snail and slug. In addition, β‐catenin and Tcf/Lef families can bind to the E‐box region of CDH‐1 gene promoter, leading to downregulation of E‐cadherin expression upon binding.^29^ However, the precise mechanisms by which cadherins are necessary for collective cell migration are yet to be fully elucidated. Additionally, the cytoskeleton provides structural support and facilitates cellular movement. Various signalling pathways contribute to individual cell migration, and among these, Rho GTPases play a crucial role.^30^ Essential cellular processes, such as cell adhesion and cell proliferation, can be regulated by these small GTP binding proteins. Rho GTPases primarily function in the regulation of actin cytoskeleton assembly, and experimental evidence strongly suggests that the Rho/ROCK signalling pathway drives cytoskeleton dynamics.^31^ Rho/ROCK signal can play a crucial role in individual cell migration, as supported by numerous studies, and is also important in collective cell migration.^32^ However, it remains unclear whether Rho/ROCK signalling behaves differently in these two modes of cell migration. In the process of collective cell migration, the adhesion between cells and extracellular matrix (ECM) is more critical than the contractile ability of individual cell skeletons.^33^ Adhesions are formed at integrin junctions in the ECM, linking integrin receptors, signalling protein complexes, structural proteins and cytoskeletal proteins together, thereby regulating cell movement within the traction range most conducive to cell migration.^34^, ^35^ Cells will migrate when the forces are within the optimal transmission threshold.^36^ However, if the force is large enough to exceed the most suitable range, the cell will stop moving.^37^, ^38^ Nevertheless, the impact of traction force on collective cell migration remains unclear.

Here, we focused on the collective migration of breast cancer cells, with a specific emphasis on cellular adhesion and cytoskeleton. We demonstrated that Notch‐1 activation induced cytoskeletal remodelling, strengthened intercellular junctions and cell‐ECM adhesion. These mechanisms collectively inhibit the migratory behaviour of breast cancer cells, highlighting the pivotal role of Notch‐1 signalling in collective breast cancer cell migration and providing novel insights into the mechanisms governing cell–cell junctions and intracellular forces during this process.

## 2 MATERIALS AND METHODS

### 2.1 Antibodies and reagents

The subsequent antibodies were utilized: phospho‐ILK (p‐ILK; Thr173), Rho, ILK, paxillin, ROCK, β‐catenin (Abcam, USA); E‐cadherin, GSK‐3β, phospho‐GSK‐3β (p‐GSK‐3β; Ser9), MYPT1, phpspho‐MLC (p‐MLC; Ser19) (Cell Signalling Technology, USA); The subsequent reagents were utilized: Phalloidin‐iFluor 647 conjugate; Estradiol valerate (Selleck, USA); Matrigel (Corning; USA); GAPDH (Servicebio, China).

### 2.2 Cell culture

MCF‐7 cells (Catalogue number: SCSP‐531) were procured from National Collection of Authenticated Cell Cultures (Shanghai, China). Cells were cultured in minimal essential medium (MEM) composed of 1% Glutamax (Gibco, USA), 1% non‐essential amino acids (Gibco, USA), 1% sodium pyruvate (Invitrogen, USA), 0.01 mg/mL recombinant human insulin (Sigma, USA), 20% newborn calf serum (Thermo Scientific, USA), and 1% penicillin and streptomycin in a standard humidified culture incubator (5% CO_2_) at 37°C.

### 2.3 Generation of MCF‐7 Notch‐1 knockdown and overexpression cell lines

The Vector, overexpressing Notch‐1 intracellular domain (NICD), scrambled short hairpin RNA (Sc.shRNA) and Notch‐1 short hairpin RNA (shRNA) lentivirus constructs were fabricated by Cyagen Biosciences, and the lentivirus transfection methods were conducted in accordance with the manufacturer's instructions. Following the transduction, puromycin (Genechem, Shanghai) at the concentration of 1 μg/mL was added to the cell culture medium to screen resistant cells, so as to obtain a stable transfected cell line. Western blotting analysis and immunofluorescence staining were used to verify the establishment of cell line. The cell lines were denoted as Vector, NICD, Sc.shRNA and Notch‐1 shRNA, respectively.

### 2.4 Cell migration assay

In order to evaluate cell migration, the Ibidi silica gel insert (Ebdi, Germany) was used and cells were implanted in two chambers with a cell density of 5 × 10^5^ cells/ml. After 18 h, the cells were basically adhered to the wall and covered, then carefully removed the insert, and 48 h later, the image was taken using the Nikon Eclipse TI2 microscope (Japan).

### 2.5 Time‐lapse microscopy

Time‐lapse microscopy was employed to evaluate the motility of MCF‐7 cells cultured in MEM medium. Images were captured every 30 min for a duration of 5 h, utilizing the definite‐focus function to maintain the focal plane on the cells during time‐lapse imaging. The positions of all cells were recorded and utilized to trace their movement. The wound healing rate, displacement and collective migration speed of each group were calculated using ImageJ software.

### 2.6 Fluorescence staining and confocal microscopy

Cells were fixed in 4% paraformaldehyde at room temperature for 15 min. Subsequently, they were permeabilized with 0.1% Triton X‐100 for 10 min and blocked with 5% bovine serum albumin (BSA) for 1 h at 37°C. The cells were incubated with p‐ILK (Mouse, 1:200), ILK (Rabbit, 1:500), Rho (Mouse, 1:250), Paxillin (Rabbit, 1:250), ROCK (Rabbit, 1:250), E‐cadherin (Mouse, 1:200), GSK‐3β (Rabbit, 1:400), p‐GSK‐3β (Rabbit, 1:400), MYPT1 (Rabbit, 1:200), and β‐catenin (Rabbit, 1:250) at 4°C overnight. Subsequently, they were incubated with secondary antibodies conjugated to Alexa Fluor 594 and Phalloidin‐iFluor 647 Conjugate for 1 h at 37°C. The samples were washed with phosphate‐buffered saline (PBS), and the cell nuclei were stained with 4′,6‐diamidino‐2‐phenylindole (DAPI). Fluorescence images were acquired using an inverted fluorescence microscope (ZEIS LSM800).

### 2.7 Western blot analysis

After washing the cells with cold PBS, the cells were lysed with RIPA lysis buffer (Beyotime, China). In the WB device, proteins were separated by sodium dodecyl sulfate‐PAGE and transferred to polyvinylidene fluoride membrane (Millipore, USA). The membranes were blocked with 5% non‐fat dry milk (or 3% BSA for tyrosine phosphorylation blots) in TBST buffer (10 mM Tris–HCl, 100 mM NaCl, and 0.1% Tween 20) for 1 h at room temperature before incubation with the following primary antibodies: Paxillin (Rabbit, 1:1000), Rho (Mouse, 1:2500), ROCK (Rabbit, 1:2000), ILK (Rabbit, 1:5000), p‐ILK (Mouse, 1:1000), β‐catenin (Rabbit, 1:5000), E‐cadherin (Mouse, 1:1000), p‐MLC (Rabbit, 1:1000), MYPT1 (Rabbit, 1:1000), GSK‐3β (Rabbit, 1:1000), p‐GSK‐3β (Rabbit, 1:1000) and GAPDH (Rabbit, 1:1000). After washing off the unbound first antibody, the second antibody of the same species was incubated in a shaker at room temperature for 2 h. The membranes were rinsed three times with TBST, and immunoreactive signals were detected utilizing the Luminol Reagent for western blotting (Beyotime, China), as per the manufacturer's guidelines.

### 2.8 Cell fractionation

A nuclear‐cytosolic protein isolation kit (Boster Biological Technology) was utilized to obtain cytosolic and nuclear fractions of MCF‐7 cells, which depends on the different solubility of various cell parts in special detergents, following the instructions.

### 2.9 In vivo xenograft model

Six‐week‐old Balb/c female nude mice were randomized into four groups (*n* = 3): Vector (Vector cell line injected), NICD (NICD cell line injected), Sc.shRNA (Sc.shRNA cell line injected), Notch‐1 shRNA (Notch‐1 shRNA cell line injected). For orthotopic injections, the cells (5 × 10^6^ cells per mouse) mixed with Matrigel (100 μL) were injected into the mammary fat pads of mice. Estradiol valerate (2 mg/kg) was administered intramuscularly to female nude mice every week according to the instructions. Six weeks post‐orthotopic injection, we sacrificed the female nude mice, and the tumour tissue and lung tissue were stained with HE.

All injections were filtered through a syringe filter (pore size 0.22 μm) for sterilization prior to animal experiments.

### 2.10 Statistical analysis

Each experiment was conducted a minimum of three times. All the data are presented as the mean ± SEM. GraphPad Prism software version 8.0 was used for statistical analysis. Statistical analysis was performed using Student's *t*‐test or one‐way analysis of variance followed by post hoc multiple comparisons. Minimum intergroup differences were considered significant at *p < 0.05, **p < 0.01, ***p < 0.001, ****p < 0.0001.

## 3 RESULTS

### 3.1 Notch‐1 signalling activation inhibits collective cell migration of MCF‐7 in vitro

**FIGURE 1:**
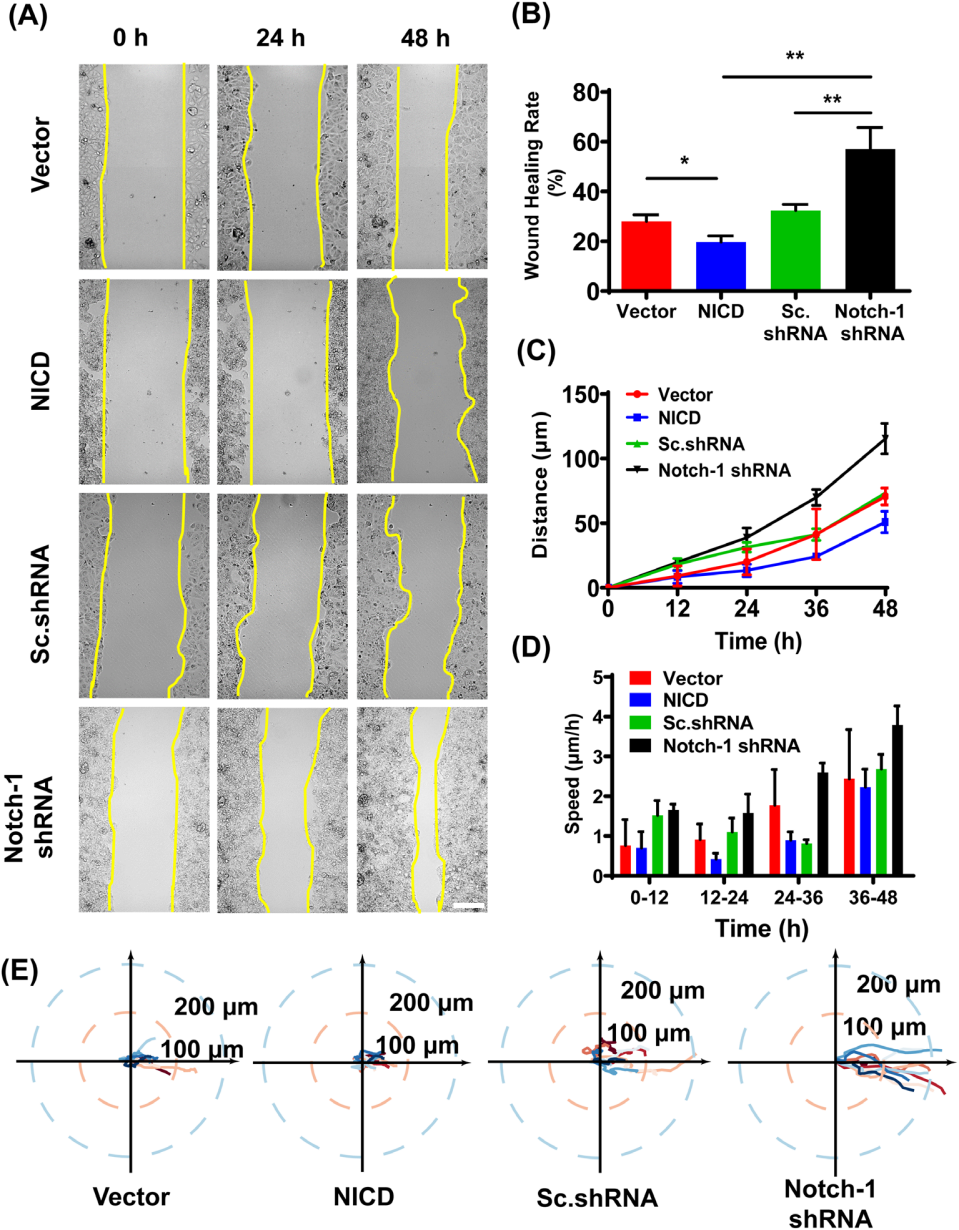
Evaluation of Notch‐1 on cell motility in MCF‐7 cells by wound healing assay. (A) Representative images of the wound healing assay in MCF7‐based cell lines in designated time points. Yellow lines indicate the leading edges. Scale bar, 200 μm. (B) The wound healing rate of migrating cell groups. (C, D) Displacement and collective migration speed of MCF‐7‐based cell lines. Data are expressed as mean ± SEM (*n* = 3, *p < 0.05, **p < 0.01). (E) The representative paths of the migration of Vector, NICD, Sc.shRNA and Notch‐1 shRNA‐transduced cell groups, as determined by tracing the nuclear centroid over a period of 48 h (*n* = 10 for each group).

To investigate the impact of Notch‐1 signalling on the collective migration of MCF‐7 cells in vitro, we established four stable cell lines through transfection: Vector, NICD, Sc.shRNA and Notch‐1 shRNA. Activation of Notch‐1 was achieved by ectopic expression of a constitutively active intracellular domain of Notch‐1, while knockdown of Notch‐1 was accomplished using shRNA. Western blot analysis was conducted to detect the expression levels of NICD in cell lines to validate the successful construction of stable transfected cell lines (Figure S1A,B). Our findings revealed that Notch‐1 activation led to a reduction in wound healing capacity, whereas Notch‐1 knockdown resulted in accelerated wound closure. In addition, we focused on the collective migration behaviour of cells, and when Notch‐1 was activated, the healing rate decreased (Figure 1A,B). Simultaneously, the overall migration speed of cells decreased (Figure 1C,D), consistent with slower wound closure of Notch‐1 activation. Additionally, we randomly selected 10 cells from each group and tracked their nuclei to characterize their specific motility paths (Figure 1E). Overall, our results indicated that activation of Notch‐1 suppressed collective cell migration.

### 3.2 Notch‐1 activation stabilizes cell–cell junctions

**FIGURE 2:**
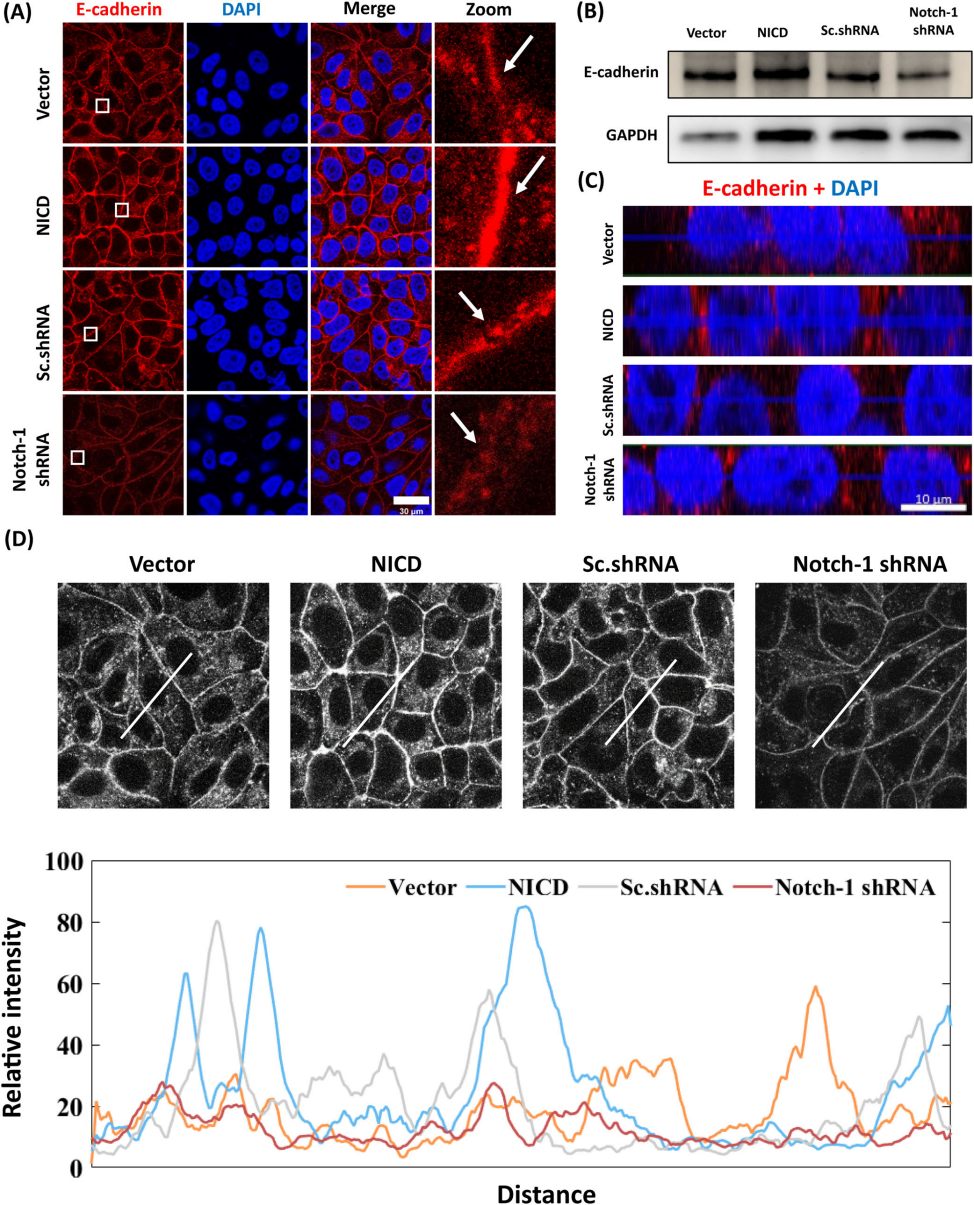
Cell–cell contacts are compromised in Notch‐1‐deficient monolayers of MCF7 cells. (A) Mature, confluent cell layers of Vector, NICD, Sc.shRNA and Notch‐1 shRNA‐transduced cell lines. Staining: E‐cadherin and DAPI. Scale bar, 30 μm. (B) Western blot with anti‐E‐cadherin antibody. (C) Lateral views of MCF7‐based cell lines stained for E‐cadherin and DNA (with DAPI). (D) Fluorescence signals were scanned along the white bar.

Collective migration relies on robust intercellular contacts, which distinguish it from individual cell migration. In order to explore the effect of Notch‐1 activation on intercellular junction, we compared the effects of Notch‐1 activation in each group using E‐cadherin staining. We observed a distinct difference, wherein Notch‐1 activation cells maintained a continuous and complete monolayer with intact junction proteins, while Notch‐1 knockdown cells exhibited disrupted junctions (Figure 2A), characterized by a punctate pattern (Figure 2A, arrows). Western blot experiments corroborated these findings (Figure 2B). Lateral views of E‐cadherin‐stained MCF‐7‐based cell lines showed that NICD had no effect on the z‐axis distribution of E‐cadherin (Figure 2C). We also assessed the fluorescence signals along a white bar, where the crest of the curve represented the fluorescence intensity at adherens junctions between cells. Notably, the NICD group exhibited a higher peak compared to the other groups (Figure 2D). These observations underscored the role of intercellular contacts in Notch‐1‐mediated migration.

### 3.3 Notch‐1 activation regulates ILK phosphorylation and GSK‐3β expressions

**FIGURE 3:**
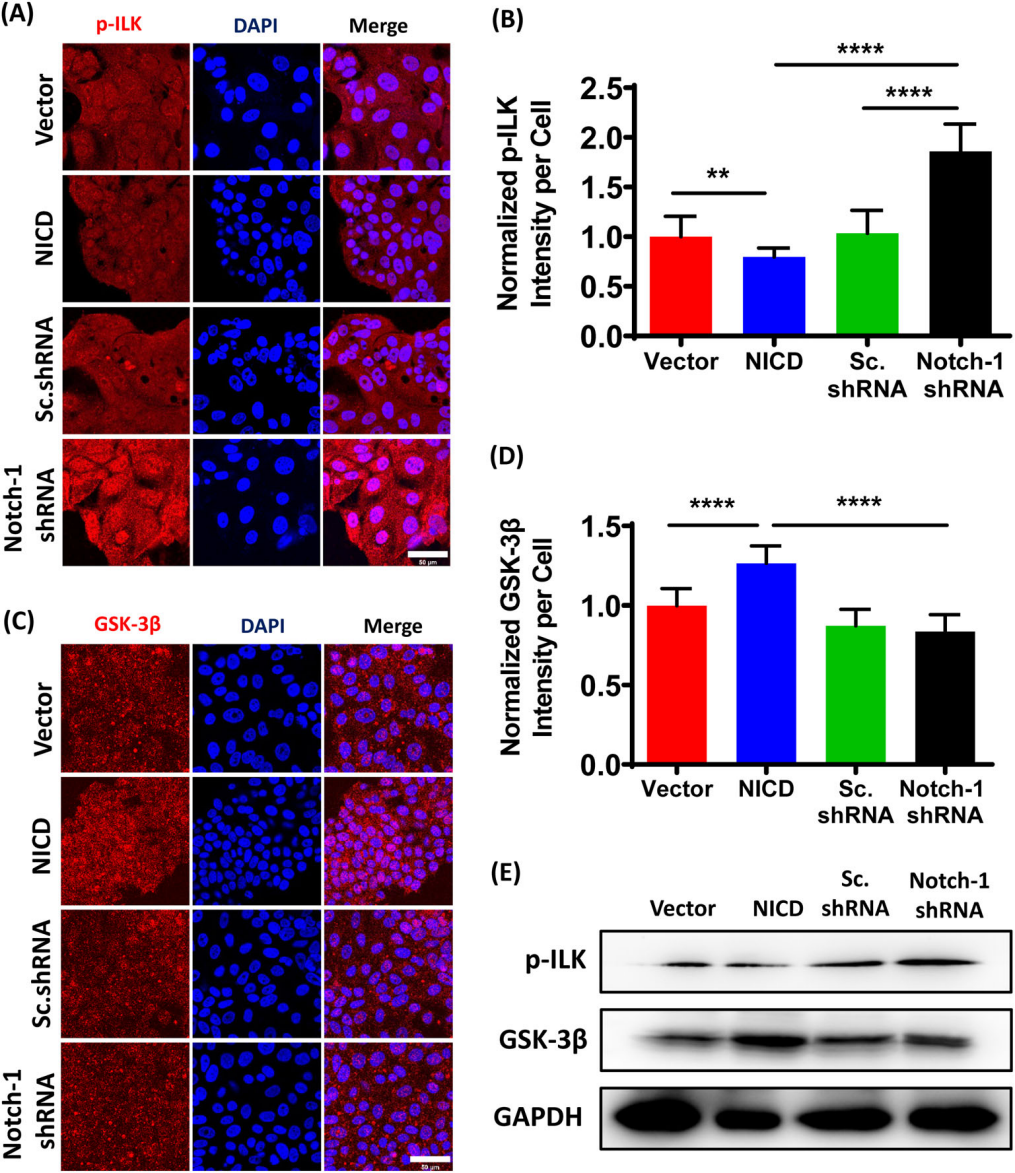
Notch‐1 activation inhibits ILK signal pathway. (A) Immunofluorescence staining showed p‐ILK (red) in MCF7‐based cell lines. Scale bar, 50 μm. (B) Spatial variation of p‐ILK fluorescent intensity normalized by cell numbers. Data are expressed as mean ± SEM (*n* = 15, *p < 0.05, **p < 0.01, ***p < 0.001). (C) Immunofluorescence staining showed GSK‐3β (red) in MCF7‐based cell lines. Scale bar, 50 μm. (D) Spatial variation of GSK‐3β fluorescent intensity normalized by cell numbers. Data are expressed as mean ± SEM (*n* = 15, ****p < 0.0001). (E) Western blot with anti‐p‐ILK and anti‐GSK‐3β antibodies.

Integrin‐linked kinase (ILK) is an important regulator in the process of cell migration, invasion, proliferation, differentiation and survival. ILK1 induces the production of vascular endothelial growth factor through downstream effector molecules such as GSK‐3β, which plays a regulatory role in tumour growth. ILK can be activated in a variety of ways, including self‐phosphorylation, binding to phosphatidylinositol‐3,4,5‐trisphosphate (PIP3) and activation of growth factor receptor tyrosine kinase. To explore the significance of Notch‐1 activation in ILK signalling, we examined the expression of ILK and ILK phosphorylation. Interestingly, Notch‐1 activation did not affect the expression of ILK in MCF‐7‐based cell lines (Figure S2A,B). However, we observed that ILK phosphorylation was remarkably inhibited by Notch‐1 activation (Figure 3A,B). Convincingly, western blot experiments confirmed the findings (Figure S3C). Our results demonstrated that Notch‐1 activation suppressed ILK phosphorylation.

Previous studies have documented the phosphorylation of GSK‐3β at Ser9 by ILK. GSK‐3 is a conserved serine/threonine kinase widely expressed in mammalian eukaryotic cells. Apart from modulating glycogen synthase (GS) activity, GSK‐3β also regulates a variety of signal molecules and transcription factors and participates in the basic activities of cells. We investigated whether NICD influenced the kinase activity of ILK by examining the intracellular expression of GSK‐3β in the four stable transfected cell lines using immunofluorescence Our results indicated that GSK‐3β dephosphorylation by ILK was significantly increased in cells with Notch‐1 activation(Figure 3C). Quantitative analysis further supported the enhanced GSK‐3β dephosphorylation in Notch‐1 activation cells (Figure 3D). It is worth noting that phosphorylation of Ser‐9 inhibits GSK‐3β activity, while dephosphorylation activates it. We observed a pronounced decrease in GSK‐3β phosphorylation by ILK in Notch‐1 activation cells (Figure S3A,B). Additionally, we assessed the expression of GSK‐3β through western blotting (Figure 3E), which aligned with our observations from immunofluorescence microscopy. Collectively, these findings indicate that the downstream signal pathway of ILK is regulated by Notch‐1 activation.

### 3.4 Notch‐1 activation enhanced E‐cadherin expression through the GSK‐3β/β‐catenin pathway

**FIGURE 4:**
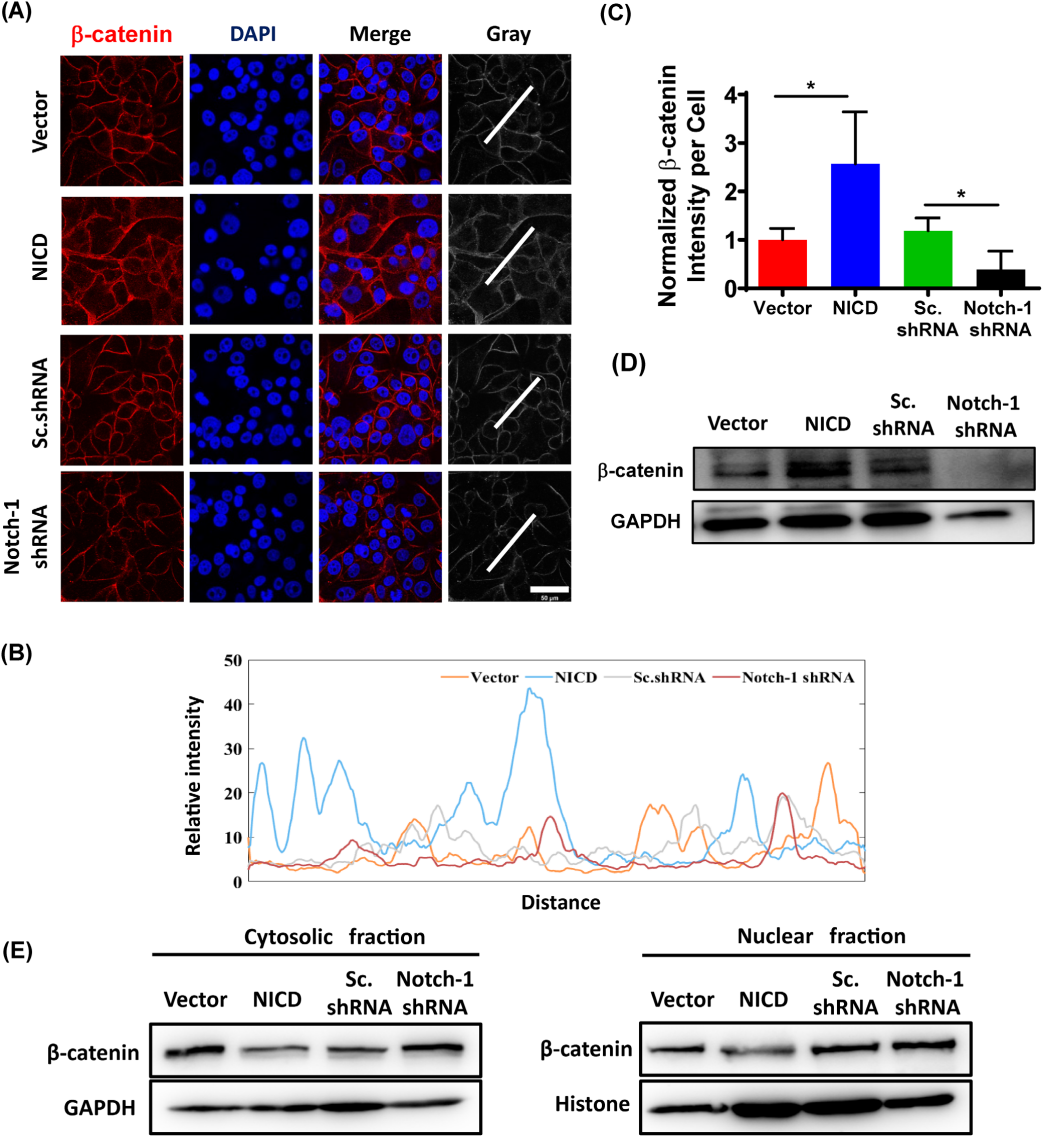
Effect of Notch‐1 shRNA on β‐catenin reduction. (A) Immunofluorescence staining showed β‐catenin (red) in MCF‐7‐based cell lines. Scale bar, 50 μm. (B) Fluorescence signals were scanned along the white bar. (C) Spatial variation of β‐catenin fluorescent intensity normalized by cell numbers. Data are expressed as mean ± SEM (*n* = 4, *p < 0.05). (D) Western blot with anti‐β‐catenin antibodies. (E) Determination of the content of β‐catenin in the cytosolic fraction and nuclear fraction.

Multifunctional carcinogenic β‐catenin can be degraded by active GSK‐3β. Analysis of images demonstrated an increase in β‐catenin expression on cellular membranes upon activation of Notch‐1 (Figure 4A–C). In line graphs, fluorescence plots are plotted along the white lines (Figure 4B). Moreover, the expression level of total β‐catenin activated by Notch‐1 can be judged by Western blotting analysis (Figure 4D). However, a fractionation experiment indicated a decrease in the amounts of cytosolic and nuclear β‐catenin upon Notch‐1 activation (Figure 4E), which contrasted with the observations of immunofluorescence microscopic images showing an increase in the membrane fraction. These findings demonstrated that Notch‐1 activation enhanced GSK‐3β activity by inhibiting ILK phosphorylation and β‐catenin expression levels in the cytosol, which hindered β‐catenin localization to the nucleus and subsequently reduced E‐cadherin expression.

### 3.5 RhoA/ROCK pathway is involved in Notch‐1‐mediated regulation of collective cell migration

**FIGURE 5:**
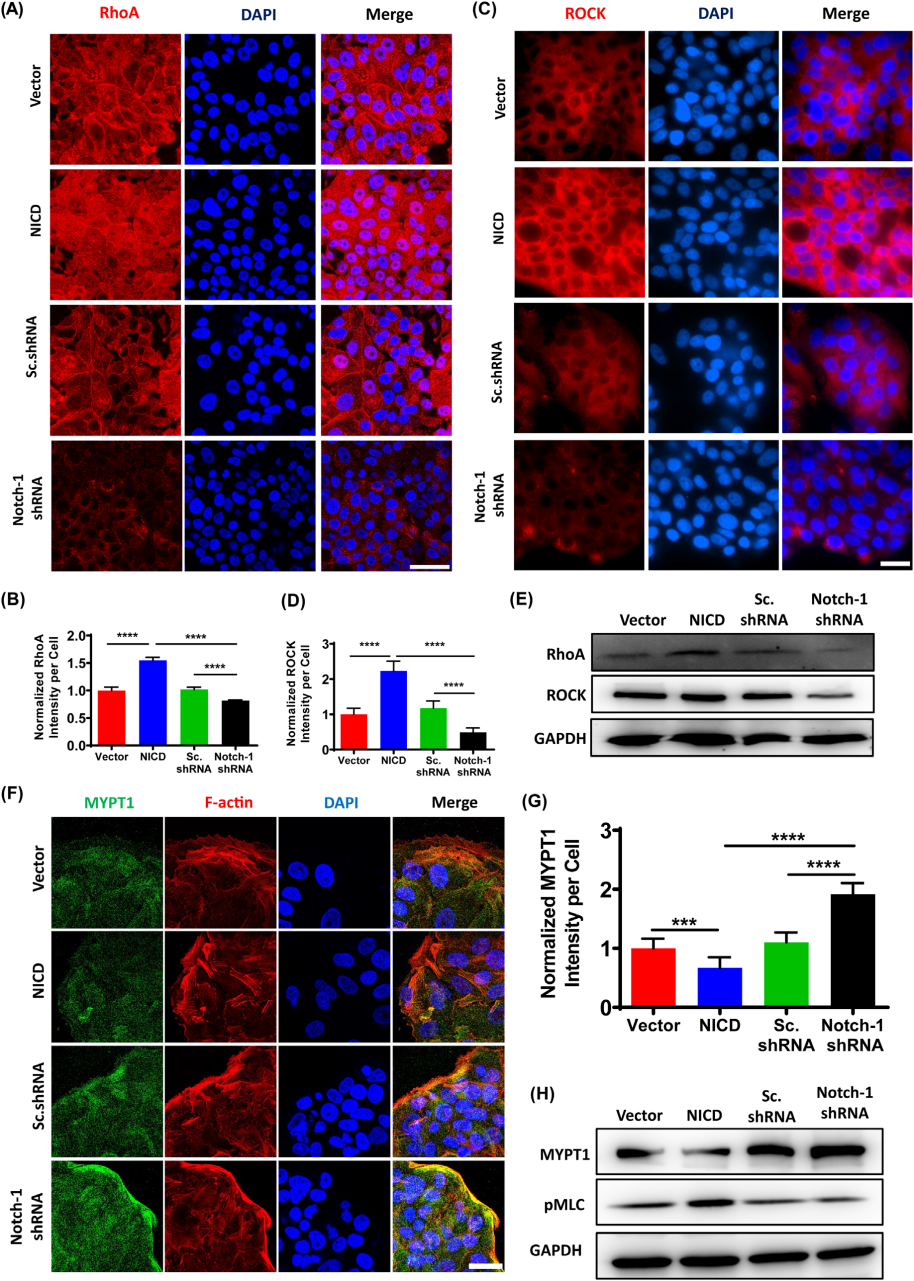
NICD mediates Rho‐ROCK signal pathway. (A) Immunofluorescence staining showed RhoA (red) in MCF7‐based cell lines. Scale bar, 50 μm. (B) Spatial variation of RhoA fluorescent intensity normalized by cell numbers. Data are expressed as mean ± SEM (*n* = 5, ****p < 0.0001). (C) Immunofluorescence staining showed ROCK (red) in MCF7‐based cell lines. Scale bar, 50 μm. (D) Spatial variation of ROCK fluorescent intensity normalized by cell numbers. Data are expressed as mean ± SEM (*n* = 15, ****p < 0.0001). (E) Western blot with anti‐RhoA and anti‐ROCK antibodies. (F) Immunofluorescence staining showed MYPT1 (green) in MCF7‐based cell lines. Scale bar, 50 μm. (G) Spatial variation of MYPT1 fluorescent intensity normalized by cell numbers. Data are expressed as mean ± SEM (*n* = 10, ****p < 0.0001). (H) Western blot with anti‐MYPT and anti‐pMLC antibodies.

Collective migration requires not only the reorganization of the cytoskeleton within a single cell, but also the adhesion mechanism in order to coordinate the movement of corresponding objects between cells and between cells and substrates. The regulation of RhoA/ROCK signal pathway is regulated in two aspects in the cytoskeleton, one is the contraction of myosin, the other is the formation of stress fibres, regulating intracellular stress levels by controlling myosin light chain phosphorylation, and influencing collective cell migration by modulating adhesion. To further elucidate the impact of Notch‐1 activation on collective cell migration, we examined whether Notch‐1 activation regulates the RhoA/ROCK pathway. Our findings revealed that the group with Notch‐1 activation exhibited elevated RhoA and ROCK activity (Figure 5A–D), and western blotting experiments confirmed the findings (Figure 5E). As actomyosin contractility and the RhoA/ROCK pathway are essential for cell migration, we investigated the expression of myosin phosphatase target subunit (MYPT1), which regulates dephosphorylation of myosin light chain (rMLC). Indeed, Notch‐1 activation decreased the level of MYPT1 (Figure 5F,G) and increased the level of phosphorylated rMLC (pMLC) (Figure 5H). These results indicate that Notch‐1 activation induces the RhoA/ROCK pathway, leading to actomyosin contraction.

### 3.6 Notch‐1 activation promotes cell‐substrate adhesion and Actin stress fibres formation

**FIGURE 6:**
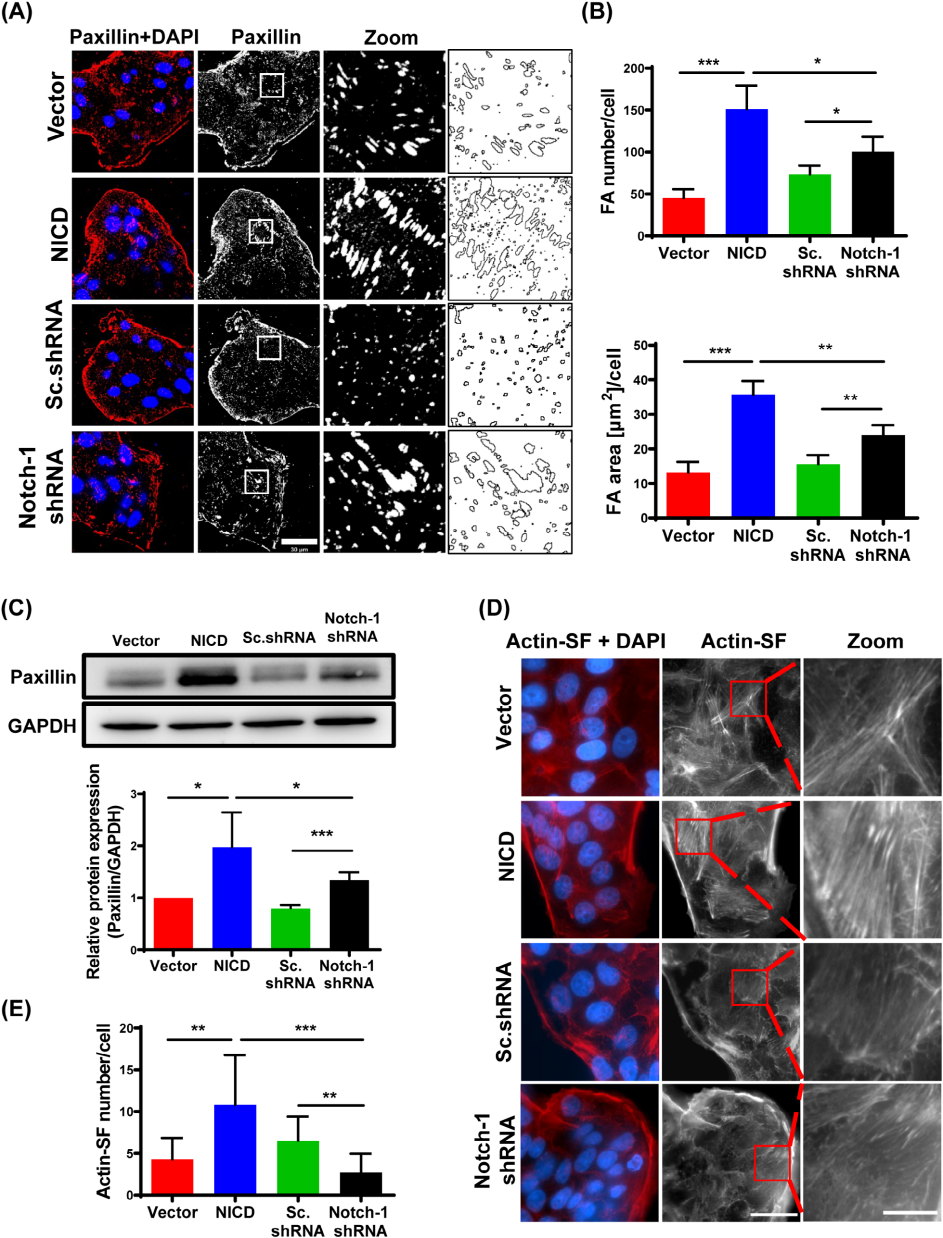
NICD affects cell‐substrate adhesion and Actin cytoskeleton organization in MCF7 cells. (A) Paxillin staining. Insets show zoomed versions of boxed areas, along with examples of ImageJ‐generated FAs count. Scale bar, 30 μm. (B) Differences in FAs number and area per cell in the studied cell lines. The measurements were performed for ∼50 cells per cell line (four independent fields of vision, 63× objective). Data are expressed as mean ± SEM (*n* = 4, *p < 0.05, **p < 0.01, ***p < 0.001). (C) Western blot with anti‐paxillin antibodies. Right panel, expression levels of the protein in each group were quantified. Data are expressed as mean ± SEM (*n* = 4, *p < 0.05, ***p < 0.001). (D) Comparison of actin stress fibres at the basal region of the cell. (E) Statistics on the number of actin stress fibre (SFs). Data are expressed as mean ± SEM (*n* = 10, **p < 0.01, ***p < 0.001).

Both cell‐substrate adhesions and cell–cell adhesion play crucial roles in the regulation of cellular migration, particularly during coordinated movement of epithelial cells. Hence, the impact of Notch‐1 on adhesions was examined. Focal adhesions were labelled with paxillin, and the size and number of local adhesions in four groups were calculated. The results showed that the number and area of focal adhesion in Notch‐1 activated group were significantly more than those in other groups. In Notch‐1‐inactivated cells, the number and size of attachment spots were also greater than those in the vector and Sc.shRNA group (Figure 6A,B). Meanwhile, this result was verified in western blot analysis (Figure 6C). Since activated Notch‐1 leads to the production of more and larger adhesive spots, the total adhesion area of Notch‐1‐activated cells increases, thereby significantly enhancing cell‐substrate adhesion. These adhesions not only impede cell migration but also necessitate overcoming contractile forces during collective cell migration. The increased size of focal adhesions in Notch‐1 activation is consistent with the increased formation of actin fibres, which anchor to focal adhesions. F‐actin organization at the basal plane was compared among the four groups, revealing more prominent actin fibres in the Notch‐1 activation group (Figure 6D,E). These correspond to the changes of monolayer tissue.

### 3.7 Notch‐1 activation inhibits the tumour metastasis in vivo

**FIGURE 7:**
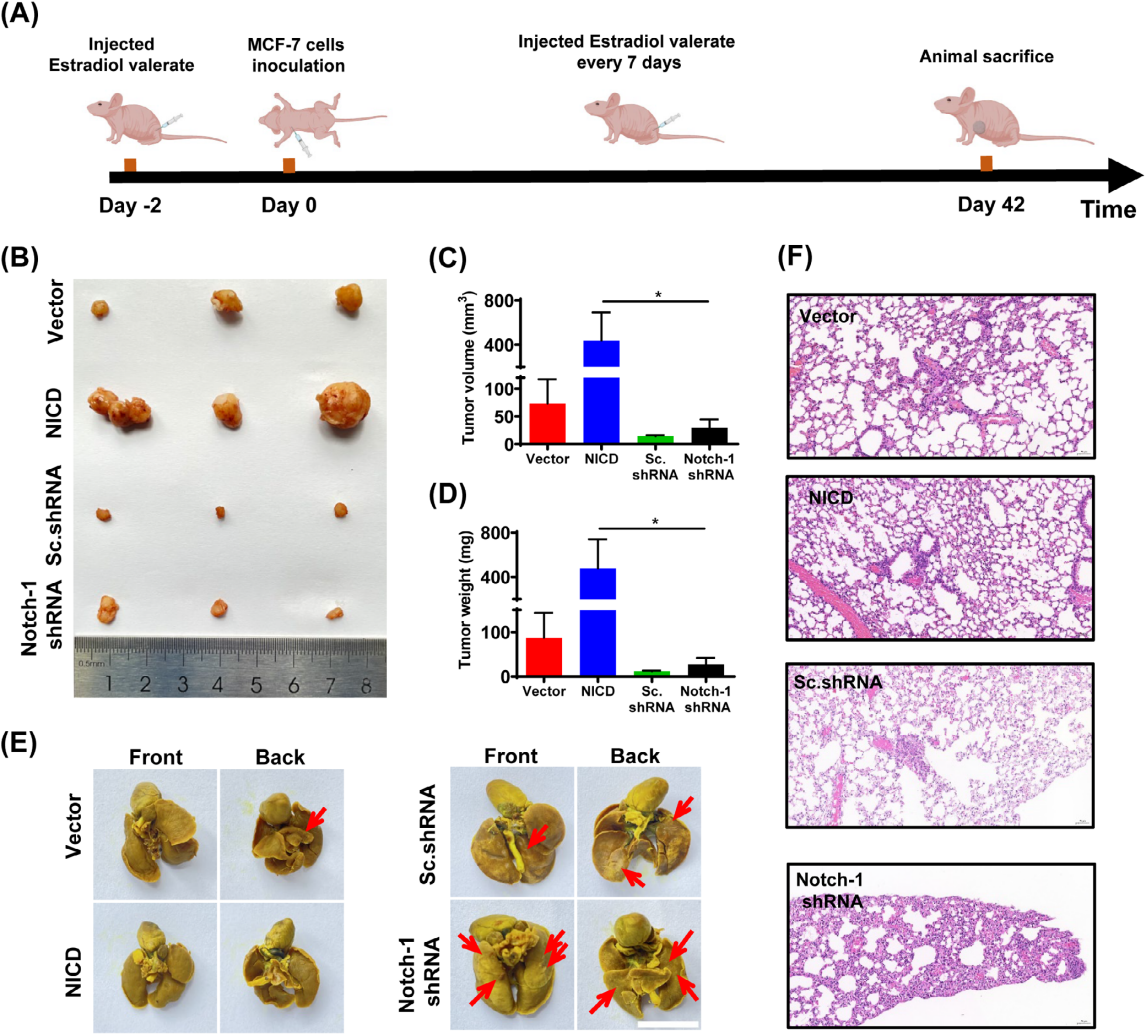
Notch‐1 activation inhibits the tumour malignancy in vivo. (A) The schematic process for tumorigenic mice of MCF‐7 model. (B) Tumour tissue was observed and collected from Balb/c nude mice by MCF‐7 with different degrees of NICD expression. (C) Ex vivo volume of tumours at the end of study. (D) Ex vivo weights of tumours at the end of study. Data are expressed as mean ± SEM (*n* = 3, *p < 0.05). (E) Lung tissues *were* fixed in Bouin's fixative. Arrows indicate metastatic tumour foci in mouse lung. Scale bar, 1 cm. (F) Representative images showing the lung nodules of sections. Lung sections were fixed and then stained with haematoxylin and eosin. Scale bar, 50 μm.

Female nude mice were employed to investigate the effects of varying degrees of Notch‐1 activation on the migration of tumour collective cells. A cell line‐derived xenograft model was established by injecting MCF‐7 cells expressing different levels of Notch‐1 into the mammary fat pad of female nude mice (Figure 7A). The mice were euthanized on the 42nd day of the experiment, and the volume and weight of the resected tumour tissue were measured and counted. Tumours in the Notch‐1 activation group exhibited significantly greater size and weight compared to the other groups, indicating that Notch‐1 activation promoted orthotopic tumour growth in vivo (Figure 7B–D). Given that a tumour has high metastatic potential, the lungs of mice were prepared to observe tumour foci formation (Figure S4C). The resected lung was fixed with Bouin's solution, and the lung tissue was stained with hue. The staining results showed that the number of pulmonary metastases decreased after Notch‐1 activation (Figure 7E,F). This suggests that Notch‐1 activation promoted orthotopic tumour growth while inhibiting metastatic progression. Additionally, immunofluorescence analysis demonstrated a significant increase in the expression of E‐cadherin and β‐catenin proteins, associated with cell–cell junctions, upon Notch‐1 activation. These experimental results and cell experiments confirm each other (Figure S4A,B).

## 4 DISCUSSION

**FIGURE 8:**
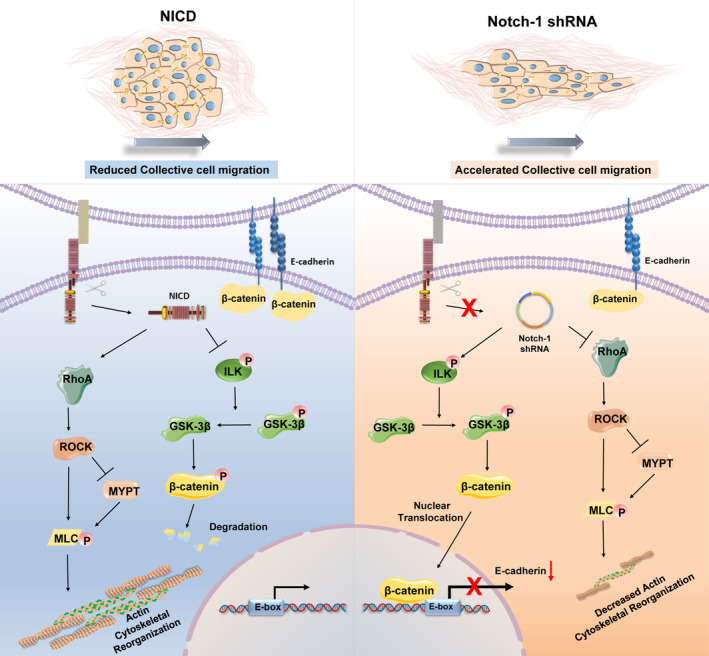
Schematic representation of the signalling pathway regulating collective cell migration. Notch‐1 activation (NICD) inhibits collective cell migration by promoting high intracellular force and strong cell–cell adhesion. Notch‐1 activation leads to GSK‐3β activity by inhibiting ILK phosphorylation, β‐catenin is degraded and cannot enter the nucleus, resulting in higher expression of E‐cadherin and tighter intercellular connections. Additionally, Notch‐1 stimulates the Rho/ROCK pathway, which enhances adhesions between cells and the ECM and ultimately impedes collective cell migration.

Notch‐1 plays an important role in tumour progression. It can regulate cell proliferation and differentiation to mediate tumorigenesis.^14^, ^39^ To investigate the contribution of Notch‐1 signalling to collective cell migration in breast cancer, we utilized a stable transfection model with four distinct cell lines (Vector, NICD, Sc.shRNA and Notch‐1 shRNA). In this study, we proved that Notch‐1 activation can activate downstream signal pathways induced cytoskeletal remodelling, strengthening of intercellular junctions and cell‐ECM of adhesion, which hindered collective cell migration (Figure 8). These results highlight that Notch‐1 signal plays a key role in the collective migration of cancer cells, and provide new insights into the regulation of cell–cell connection and cellular force in this process.

Interestingly, our previous findings demonstrated that Notch‐1 signalling typically accelerates individual cell migration.^40^, ^41^ Opposite to individual cell migration, Notch‐1 signalling actually inhibits collective cell migration, and exhibits directional polarization and coordinated movement. Collective migration not only involves the reorganization of the cytoskeleton of individual cells, but also requires the mechanism of cell adhesion, which can respond to the physical coordination in population migration. During collective cell migration, the reorganization of the cytoskeleton plays a crucial role in generating contractile forces.^42^ Cell traction forces primarily originate from leader cells and the cytoskeleton, which is closely linked to the leader cells. It can sense and respond to these forces by triggering cytoskeletal reorganization,^43^ thereby coordinating migratory polarity and migration rate of collective cell migration. However, high contractility can increase both the intracellular force of leader cells and the force of follower cells, which can lead to abnormal force distribution and slow down collective cell migration. Migration of cells requires myosin tension. However, coordinating the three steps of myosin treadmilling, leading edge adhesion formation and trailing edge detachment is a challenge that needs to be addressed in collective cell migration.^44^ In NICD cells, myosin II activity is confirmed to be enhanced by the myosin light chain phosphorylation assay. To further elucidate the molecular mechanisms underlying Notch‐1 signalling mediated regulation, we studied the involvement of RhoA/ROCK pathways in individual cells that control myosin contraction and stress fibre formation. We found that RhoA and ROCK can coordinate the dynamics of actin networks to achieve the optimal function of actin, which leads to the migration behaviour of single cells. In individual cell migration, inhibition of RhoA or ROCK decreases migration. Notch‐1 has been previously shown to influence RhoA activity in individual cells, and our findings suggest that Notch‐1 also impacts RhoA activity in MCF‐7 cells, although the effect is moderate. For collective cell migration, too high RhoA activity reduces migration. This suggests that the RhoA/ROCK pathway is involved in Notch‐1‐mediated regulation, but it may not be the unique mechanism. Future research should aim to determine whether Notch‐1 modulates GTPase activity. E‐cadherin serves as a hallmark protein for adhesive connections. Generally, loss of E‐cadherin leads to cell dispersion into single, motile cells.^45^ However, some types of tumour invasion usually occur through the collective migration of cells, which indicates that invasive and metastatic tumour cells do not always lack cell‐to‐cell connections.^46^ The cytodomain of E‐cadherin binds to β‐catenin, which recruits α‐catenin that interacts with actin filaments. β‐catenin is widely distributed in cells, not only on the cell membrane but also in the cytoplasm and nucleus. In our study, the experimental results demonstrated the highest overall expression of β‐catenin and β‐catenin at the cell membrane in the Notch‐1 activated group. However, the expression of β‐catenin in the cytoplasm and nucleus was the lowest in this group. However, the expression of β‐catenin in the cytoplasm and nucleus are the lowest in this group. This is probably because Notch‐1 activation prevented cytoplasmic free β‐catenin from translocating to the nucleus due to degradation by the GSK‐3β complex by activating GSK‐3β, and E‐cadherin expression increased which led to tight intercellular junctions.

It is widely acknowledged that activated glycogen synthase kinase‐3 beta (GSK‐3β) can establish complexes with beta‐catenin and enhance its degradation. Integrin‐linked kinase (ILK), the upstream kinase of GSK‐3β, can be activated via phosphorylation.^47^ The activity of GSK‐3β is predominantly regulated through phosphorylation. Protein kinase B (PKB/Akt), protein kinase A (PKA), and mitogen‐activated protein kinase‐activated protein (MAPKAP) kinase‐1 (p90rsk) inhibit GSK‐3β by phosphorylating serine 9 of GSK‐3β. Protein phosphatase (PP) 1 dephosphorylates serine 9 of GSK‐3β, thereby increasing GSK‐3β activity. GSK‐3β is also controlled through the formation of distinct protein complexes. GSK‐3β establishes complexes with axin, adenomatous polyposis coli (APC), casein kinase 1 (CK1), and beta‐catenin, which facilitate the phosphorylation of beta‐catenin in the cytoplasm at threonine 41, serine 37, and serine 33, resulting in its ubiquitination and degradation.^48^ In our investigation, within the group exhibiting active Notch‐1 signalling, cytoplasmic beta‐catenin undergoes degradation mediated by activated GSK‐3β, leading to the highest expression of E‐cadherin.

Highly conserved Notch signalling pathways exist widely in cells and determine the fate of cells.^39^ The activation of Notch is closely related to cell proliferation.^33^ Previous studies have found the Notch‐1 deficiency prevents the differentiation of ventricular like cardiomyocytes and leads to defects in cardiomyocyte proliferation. These can all lead to left ventricular hypoplasia in congenital heart disease.^49^ In Figure 7, the results demonstrated that tumours in the Notch‐1 activated group were significantly larger and heavier than those formed by other groups, which indicated that Notch‐1 activation promoted orthotopic tumour growth in vivo. Notch‐1 activation could promote orthotopic tumour growth but cause lower tumour metastasis, which is an interesting direction for the future studies. Interestingly, we discovered that the tumours were smaller in the Sc.shRNA group than in the other groups. This may be because Sc.shRNA may interfere with growth modification genes.

Cell collective migration is a complex cellular activity, which involves the synergistic effect of multiple cells. Our results indicate that Notch‐1 activated cells (NICD group) exhibit enhanced cell–cell contacts and increased contractility, which leads to slower collective migration. By elucidating the molecular mechanisms of collective cell migration regulated by Notch, we hope to provide valuable insights for the development of targeted therapies to inhibit tumour metastasis. Understanding the intricate relationship between Notch signalling and collective cell migration has the potential to revolutionize cancer treatment strategies and improve patient outcomes.

## AUTHOR CONTRIBUTIONS

**Yixi Zhang**: Writing—original draft, project administration, methodology, investigation, formal analysis, data curation. **Xiang Qin**: Writing—review & editing, project administration, methodology, investigation. **Ronghua Guo**: Project administration, investigation. **Xiyue Sun**: Investigation. **Zihan Zhao**: Project administration, investigation. **Hanyu Guo**: Resources. Meng Wang: Resources. **Shun Li**: Resources. **Tingting Li**: Resources. **Dong Lv**: Visualization, Funding acquisition. **Yiyao Liu**: Writing—review & editing, supervision, funding acquisition. All authors have read and approved the final version of the manuscript and agree to be accountable for all aspects of the work.

## CONFLICT OF INTEREST STATEMENT

The authors declare no conflicts of interest.

## Supporting information


**Figure S1.** NICD expression assay. (A) Four stable transfected cell lines were inoculated into six well plates and cultured for 48 h. Bright field and fluorescence photography were carried out by fluorescence inverted microscope. Scale bar, 200 μm. (B) The NICD expression was detected by western blotting.
**Figure S2.** The total expression of ILK is not affected by NICD. (A) Immunofluorescence staining showed ILK (red) in MCF7‐based cell lines. Scale bar, 50 μm. (B) Spatial variation of ILK fluorescent intensity normalized by cell numbers. Data are expressed as mean ± SEM (*n* = 15).
**Figure S3.** NICD decreases GSK‐3β phosphorylation. (A) Immunofluorescence 4 staining showed p‐GSK‐3β (red) in MCF7‐based cell lines. Scale bar, 50 μm. (B) Spatial variation of p‐GSK‐3β fluorescent intensity normalized by cell numbers. Data are expressed as mean ± SEM (*n* = 15, ****p < 0.0001). (C) Western blot with anti‐ILK and anti‐p‐GSK‐3β antibodies.
**Figure S4.** Representative pictures of E‐cadherin and β‐catenin staining (immunofluorescence) in vivo. (A) Immunofluorescence staining showed E‐cadherin (red) in tumours. (B) Immunofluorescence staining showed β‐catenin (green) in tumours. Scale bar, 50 μm. (C) Lung tissues of mice injected with four stable transfected cell lines.

## Data Availability

The data that support the findings of this study are available from the corresponding author upon reasonable request.
