# Supplementary material for: Notch‐1 regulates collective breast cancer cell migration by controlling intercellular junction and cytoskeletal organization
Source: Cell Prolif. 2024 Sep 29;58(2):e13754. doi: 10.1111/cpr.13754 (PMC11839191; doi:10.1111/cpr.13754)
Supplement: Supplementary file 1 — Figure S1. NICD expression assay. (A) Four stable transfected cell lines were inoculated into six well plates and cultured for 48 h. Bright field and fluorescence photography were carried out by fluorescence inverted microscope. Scale bar, 200 μm. (B) The NICD expression was detected by western blotting. Figure S2. The total expression of ILK is not affected by NICD. (A) Immunofluorescence staining showed ILK (red) in MCF7‐based cell lines. Scale bar, 50 μm. (B) Spatial variation of ILK fluorescent intensity normalized by cell numbers. Data are expressed as mean ± SEM (n = 15). Figure S3. NICD decreases GSK‐3β phosphorylation. (A) Immunofluorescence 4 staining showed p‐GSK‐3β (red) in MCF7‐based cell lines. Scale bar, 50 μm. (B) Spatial variation of p‐GSK‐3β fluorescent intensity normalized by cell numbers. Data are expressed as mean ± SEM (n = 15, ****p < 0.0001). (C) Western blot with anti‐ILK and anti‐p‐GSK‐3β antibodies. Figure S4. Representative pictures of E‐cadherin and β‐catenin staining (immunofluorescence) in vivo. (A) Immunofluorescence staining showed E‐cadherin (red) in tumours. (B) Immunofluorescence staining showed β‐catenin (green) in tumours. Scale bar, 50 μm. (C) Lung tissues of mice injected with four stable transfected cell lines. [file CPR-58-e13754-s001.pdf]

Supplementary Information

Notch-1 regulates collective breast cancer cell migration by controlling intercellular junction and cytoskeletal organization

Yixi Zhang [1], Xiang Qin [1*], Ronghua Guo [1], Xiyue Sun [1], Zihan Zhao [1], Hanyu Guo [1], Meng Wang [1], Shun Li [1], Tingting Li [1], Dong Lv [2*], Yiyao Liu [1, 2, 3*]

[1] Department of Pharmacy, Personalized Drug Therapy Key Laboratory of Sichuan Province, Sichuan Provincial People's Hospital, and School of Life Science and Technology, University of Electronic Science and Technology of China, Chengdu, China

[2] Department of Urology, Deyang People's Hospital, Deyang, China

[3] TCM Regulating Metabolic Diseases Key Laboratory of Sichuan Province, Hospital of Chengdu University of Traditional Chinese Medicine, Chengdu, China

***Corresponding authors**:

Email address: qinxiang@uestc.edu.cn (X. Qin), lv-0919@163.com (D. Lv), *or* liuyiyao@uestc.edu.cn (Y. Liu)

Supplementary Figures and Figure Legends

Figure S1: NICD expression assay.

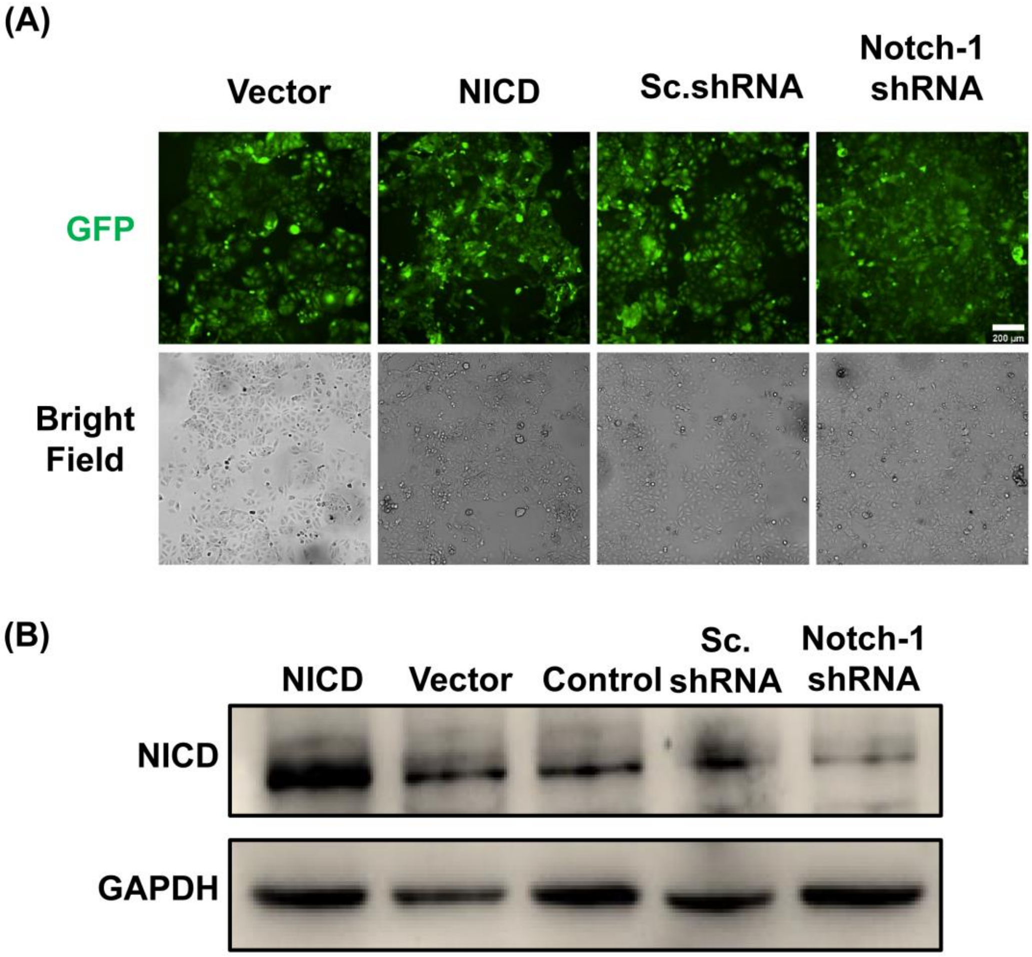

Figure S1 NICD expression assay. (**A**) Four stable transfected cell lines were inoculated into six well plates and cultured for 48 h. Bright field and fluorescence photography were carried out by fluorescence inverted microscope. Scale bar, 200 μm. (**B**)The NICD expression was detected by western blotting.

Figure S2: The total expression of ILK is not affected by NICD.

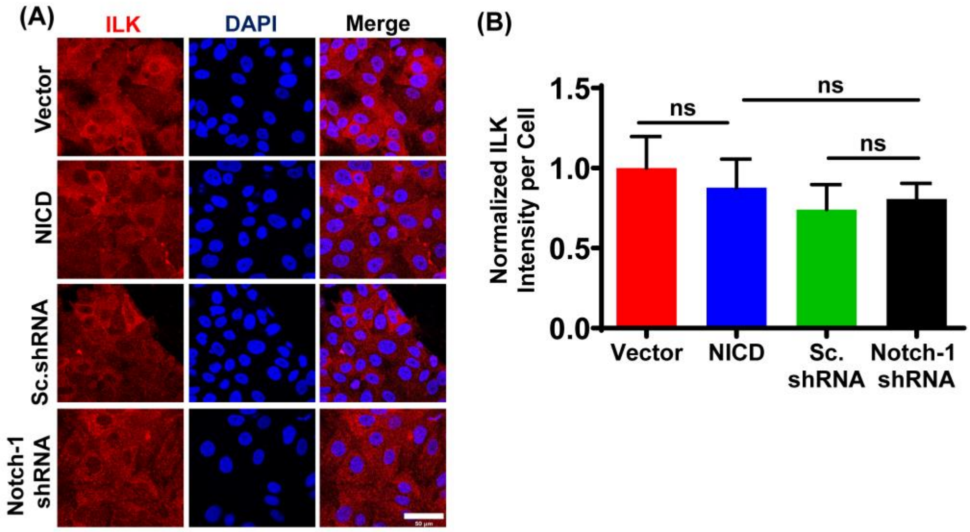

Figure S2 The total expression of ILK is not affected by NICD. (**A**) Immunofluorescence staining showed ILK (red) in MCF7-based cell lines. Scale bar, 50 µm. (**B**) Spatial variation of ILK fluorescent intensity normalized by cell numbers. Data are expressed as mean ± s.e.m. (n=15).

Figure S3: NICD decreases GSK-3β phosphorylation.

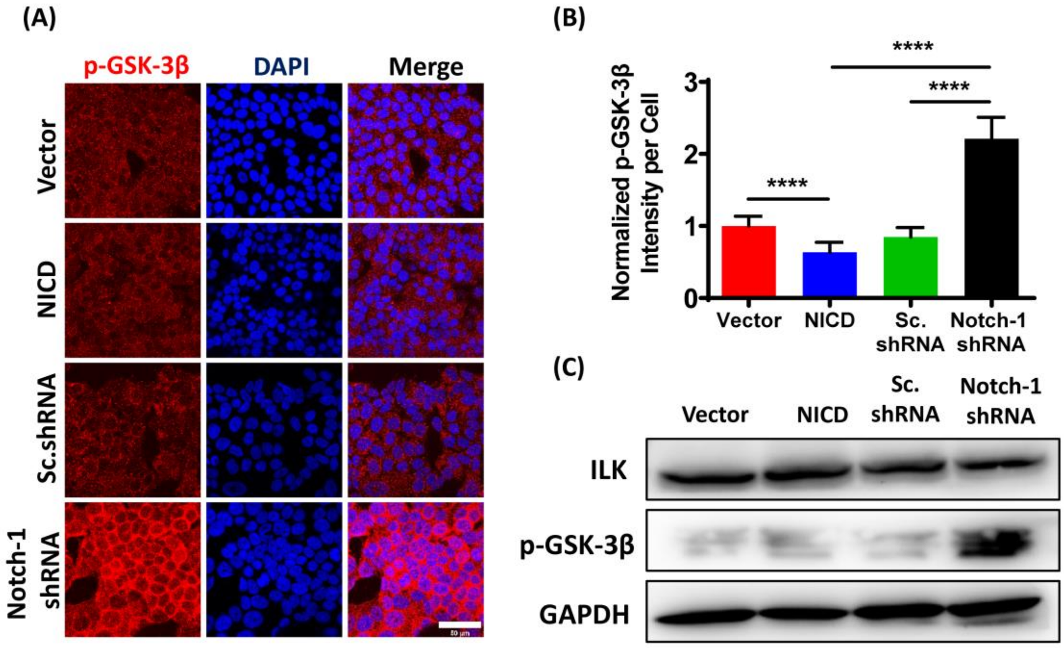

Figure S3 NICD decreases GSK-3β phosphorylation. (**A**) Immunofluorescence

staining showed p-GSK-3β (red) in MCF7-based cell lines. Scale bar, 50 μm. (**B**) Spatial variation of p-GSK-3β fluorescent intensity normalized by cell numbers. Data are expressed as mean ± s.e.m. (n=15, ****$p<0.0001$). (**C**) Western blot with anti-ILK and anti-p-GSK-3β antibodies.

Figure S4: Representative pictures of E-cadherin and β-catenin staining (immunofluorescence) *in vivo*.

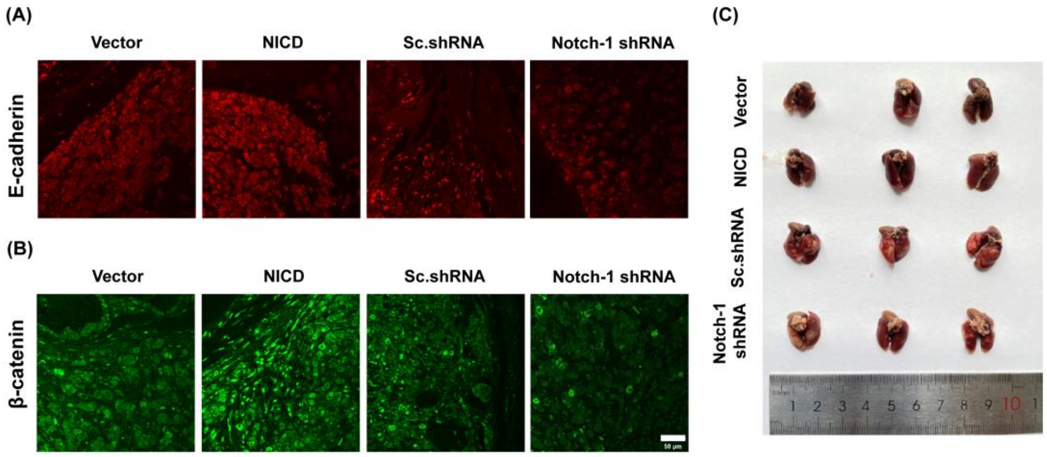

Figure S4 Representative pictures of E-cadherin and β-catenin staining (immunofluorescence) *in vivo*. (**A**) Immunofluorescence staining showed E-cadherin (red) in tumors. (**B**) Immunofluorescence staining showed β-catenin (green) in tumors. Scale bar, 50 μm. (**C**) Lung tissues of mice injected with four stable transfected cell lines.
